# The ratio of effect-based combination indexes can indicate nonapoptotic cell-death in a combined cancer therapy

**DOI:** 10.3389/fphar.2026.1737170

**Published:** 2026-01-22

**Authors:** Tianran Yu, Yuxuan Dong, Xinya Li, Tinghe Yu

**Affiliations:** 1 College of Pharmaceutical Sciences, Southwest University, Chongqing, China; 2 International Medical College, Chongqing Medical University, Chongqing, China; 3 Laboratory of Obstetrics and Gynecology, The Second Affiliated Hospital, Chongqing Medical University, Chongqing, China

**Keywords:** apoptosis, apoptosis kinetics, combination index, combined therapy, nonapoptosis

## 1 Introduction

Determining cell-death modes is critical in developing a combined cancer therapy: the combination may deactivate cells via a mode differed from that in the single regimen, or via more than one mode. Most single therapies deactivate cancer cells via the apoptosis pathway, and the apoptotic malfunction results in therapeutic resistance (Liu et al., 2025; Yue et al., 2025). Therefore, that a combined therapy can induce nonapoptotic death is a specific merit, for having a potential to combat resistance (Yu et al., 2016; Liu et al., 2022; Yu and Li, 2023; Swords et al., 2024; Moyer et al., 2025). Whether there is nonapoptotic death should be decided at an early state of a therapeutic trial.

Of those cell-death modes, apoptotic cells (i.e., the apoptosis percentage) can be directly and accurately quantified using easy methods (e.g., annexin V, terminal nick end labeling or sub-G1 assay). Indeed, the apoptosis percentage is calibrated in most therapeutic trials. However, the death percentages due to other modes (e.g., necrosis, necroptosis, ferroptosis or autophagy) can only be detected qualitatively or semi-quantitatively: nonapoptotic death is demonstrated by determining the bio-chemical biomarkers related to a specific death mode or observing a reduction in the death percentage after inhibiting a specific death mode (Hu et al., 2021; Kari et al., 2022; Liu et al., 2022; Khalef et al., 2024).

## 2 Determining nonapoptotic death based on the ratio of combination indexes

The effect-based combination index (CI) is used to assess a combined therapy (Equation 1).

$$CI=\frac{E_{A+B}}{E_{A}+E_{B}-E_{A}\times E_{B}}$$ (1)

E_A_ or E_B_ is the effect of a single therapy, and E_A+B_ is the effect of the combination. The dose of A in E_A+B_ is equal to that in E_A_, so is the dose of B in E_A+B_ and E_B_. A CI of <0.85, 0.85–1.15, or >1.15 indicates antagonism, addition, or synergy, respectively (Yu et al., 2025). CI can be calculated using the percentages of dead cells (i.e., CI_Dea_) or other effects (He et al., 2014). CI_Dea_ reflects the ultimate effect, because induction of death of cancer cells is the primary efficacy endpoint. A combined therapy can be further developed only when CI_Dea_ shows addition or synergy (He et al., 2012).

Each death mode contributes to a fraction of cell death when death results from multiple modes, and the sum is the percentage of dead cells (Equation 2). Thus, each death mode can be considered as a specific effect, i.e., having a corresponding CI (e.g., CI_Apo_ is based on the percentages of apoptotic cells). CI_Dea_ is the gross effect (Equation 3).

%death=%apoptosis+%ferroptosis+%necrosis+... (2)

$$CI_{Dea}=\sum_{i=1}^{n}\left(CI_{i}\times W_{i}\right)$$ (3)

CI_i_ is for a specific death mode, and W_i_ is the weight of that mode in overall cell death. However, the percentage of nonapoptotic death cells cannot be quantified directly and accurately, i.e., CI_i_ except CI_Apo_ cannot be directly calculated. Theoretically, other CI_i_ can be deduced by CI_Dea_ minus CI_Apo_. These suggest that the ratio of CI_Apo_ to CI_Dea_ can provide info of the cell-death modes.

Here discussions are based on these premises: the identical therapeutic manner (doses and exposure duration of drugs) is employed in both cell-death and apoptosis trials, cell death and apoptosis are calibrated at the same time point, and CI_Dea_ indicates addition or synergy.

**FIGURE 1:**
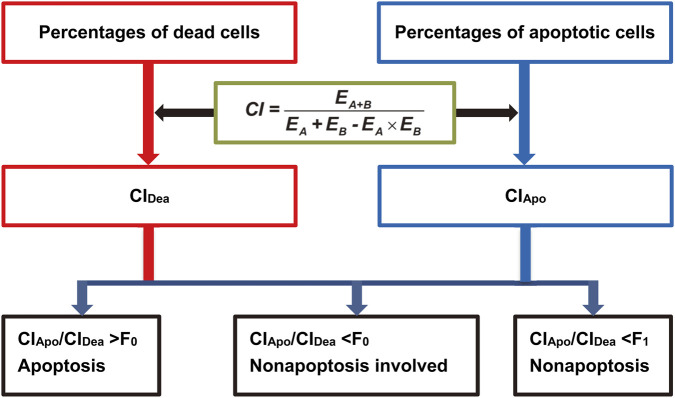
The ratio of CI_Apo_ to CI_Dea_ can indicate the occurrence of nonapoptotic death in a combined therapy. F_0_ and F_1_ are cell-type dependent; the empirical values are 0.8 and 0.4–0.5, respectively. CI_Dea_, combination index based on death percentages; CI_Apo_, combination index based on apoptosis percentages.

1. When apoptosis is the sole/determining death mode in the combined therapy, the number of dead cells is the sum of apoptotic cells. Accordingly, CI_Apo_ is nearly equal to CI_Dea_, i.e., CI_Apo_ >F_0_×CI_Dea_. F_0_ can be empirically set 0.8.
2. When there is a nonapoptotic death mode in the combination, the percentage of dead cells is partly due to that of apoptotic cells. Therefore, CI_Apo_ contributes to only a fraction of CI_Dea_, i.e., CI_Apo_ <F_0_×CI_Dea_.
3. In rare cases, the combination deactivates cells via a nonapoptotic mode although apoptosis is the mechanism for the single therapy (Ma et al., 2016; Liu et al., 2017; Silva et al., 2017). Consequently, the apoptosis percentage in the combination is not increased in comparison with the single therapy, i.e., CI_Apo_ <F_1_×CI_Dea_. F_1_ can be set 0.4–0.5 (depending on cell type) (Figure 1).

These deductions were tested in 2 datasets. The agreement was 77.6% (111/143) when using the death mode stated in the trial as the reference (κ = 0.40 [95% confidence interval: 0.23–0.57], p < 0.0001) (noticeably, data can be extracted from 39/198 papers released in 2016–2017, but from 37/308 papers released in 2022–2024). The accuracy for apoptosis was 81.4% (92/113), and that for nonapoptosis was 63.3% (19/30), respectively (Supplementary Tables S1, S2). These findings suggest that the CI_Apo_/CI_Dea_ ratio can be used to preliminarily determine the death mode in a combined therapy.

Based on the present data, an inquiry about the role of autophagy arises. Autophagy was considered a death mechanism in certain trials, but CI_Apo_>CI_Dea_ indicated that apoptosis was the determining death mode (Wang et al., 2016; Zhang et al., 2017). A reasonable interpretation is that the increase in autophagy is an accompanying response, or that autophagy is the switch to apoptosis. There arise similar concerns when necrosis is deemed as a death mechanism based on only propidium iodide-positive cells in flow cytometry but CI_Apo_ is not inferior to CI_Dea_ (Shirvalilou et al., 2024).

## 3 Improving the accuracy

CI_Dea_ has high accuracy, since available assays for cell viability are with high selectivity, specificity and stability. Therefore, the accuracy of the present method relies on accurate CI_Apo_ which is determined by accurate apoptosis percentages. The apoptosis percentage in control cells (receiving no therapy) was >10% (even >20%) in certain trials, demonstrating a high background level (Yadav et al., 2022; Zheng et al., 2023; Camero et al., 2024). Such a high background level suggests biases in apoptosis percentages in cells receiving therapies, which will distort CI_Apo_. An apoptosis percentage of <5% in control cells indicates that data are with high quality for evaluations.

One dose for each single therapy is commonly used in an apoptosis trial. In certain cases, the dose may not be the optimum one to determine apoptosis in the combination, thereby underestimating CI_Apo_ and eventually leading to misjudgments. A preferred method is to adopt 2–3 doses in each single therapy to catch the apoptosis property better, particularly when the CI_Apo_/CI_Dea_ ratio is 0.7–0.8. Doses for the apoptosis trial can be set according to the death percentage vs. dose curve, and should be up to the half-maximum effective dose. A higher dose may lead to saturation, thereby covering up apoptosis synergy (Yu et al., 2025).

**FIGURE 2:**
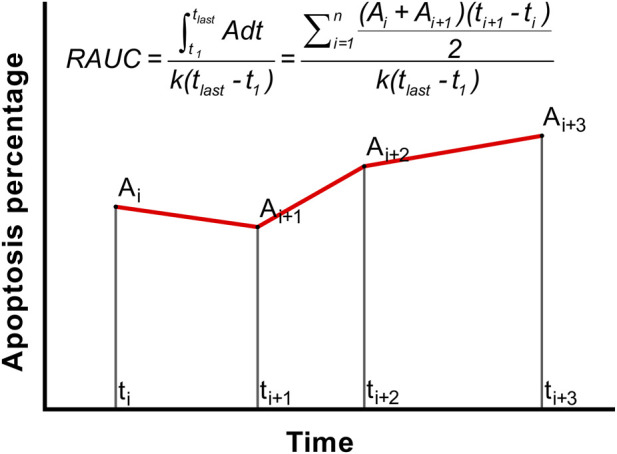
AUC is integrated using the trapezoidal method, and then RAUC is calculated. The coefficient k outlines the panorama of apoptosis, and the default value can be 1.0. AUC, area under the apoptosis percentage vs. time curve; RAUC, relative AUC.

Apoptosis does not necessarily synchronize with cell death. In certain cases, the apoptosis pattern of a regimen in the combination differs from that of being administrated alone, i.e., alterations of apoptosis percentages in two single and the combined therapies are nonsynchronous. The apoptosis percentage in the combined therapy can be lower than the actual level, thereby distorting CI_Apo_ and eventually resulting in misjudgments. The apoptosis kinetics may be a solution. Apoptosis percentages in a definite duration (t_1_–t_last_) are quantified at multiple time points. RAUC (relative area under the apoptosis percentage vs. time curve) is used to calculate CI_Apo_, where integration utilizes the trapezoidal method (Figure 2; Equations 4, 5) (Yu et al., 2024).

$$RAUC=\frac{\int_{t_{1}}^{t_{last}}Adt}{k\left(t_{last}-t_{1}\right)}=\frac{\sum_{i=1}^{n}\frac{\left(A_{i}+A_{i+1}\right)\left(t_{i+1}-t_{i}\right)}{2}}{k\left(t_{last}-t_{1}\right)}$$ (4)

$$CI_{Apo}=\frac{RAUC_{A+B}}{RAUC_{A}+RAUC_{B}-RAUC_{A}\times RAUC_{B}}$$ (5)

t_last_ should be identical to the time point of determining the cell-death percentage. A_i_ is the apoptosis percentage at the i-th time point. The coefficient k (depending on cell type) outlines the apoptosis panorama, and should be biologically and mathematically logical (i.e., RAUC complies with the rule of calculating CI). Thus, k should be 0.8–1.0, and the default value can be 1.0 (i.e., with an apoptosis percentage of 100%). CI_Apo_ based on RAUC is the preferred method when apoptosis percentages indicate that nonapoptotic death is the sole mechanism for the combination, but either single therapy deactivates cells via apoptosis. This manner can avoid false negative.

## 4 Conclusion

The CI_Apo_/CI_Dea_ ratio provides a preliminary indicator of cell-death modes. A higher ratio confirms apoptosis and a lower ratio suggests the involvement of nonapoptotic death, which can be used to assess the potential of a combined therapy. Individually optimizing F_0_ or F_1_ may be needed in specific cases. The accuracy of this method is reliant on accurate CI_Apo_ that is determined by accurate apoptosis percentages. Using RAUC to calculate CI_Apo_ can improve the accuracy when apoptosis does not synchronize with cell death.
