# Supplementary material for: The ratio of effect-based combination indexes can indicate nonapoptotic cell-death in a combined cancer therapy
Source: Front Pharmacol. 2026 Jan 22;17:1737170. doi: 10.3389/fphar.2026.1737170 (PMC12872802; doi:10.3389/fphar.2026.1737170)
Supplement: Supplementary file 1 [file DataSheet1.pdf]

Table S1. List of combination indexes (CI) and cell-death modes in released trials (2016−2017)

Trial	Cell	Treatment and effect				CI	CI ratio	Mode based on CI ratio	Mode stated in the trial
Cancer Lett			BV6	MS275	Combination				
2015,366:32	OCI-AML3	Death	0.16	0.25	0.54	1.46	1.11	Apoptosis	Apoptosis
		Apo	0.12	0.26	0.56	1.62			
	MV4-11	Death	0.19	0.24	0.62	1.61	0.90	Apoptosis	Apoptosis
		Apo	0.24	0.24	0.60	1.44			
	Molm13	Death	0.21	0.15	0.55	1.67	0.98	Apoptosis	Apoptosis
		Apo	0.23	0.16	0.57	1.64			
	NB4	Death	0.24	0.21	0.68	1.71	0.81	Apoptosis	Apoptosis
		Apo	0.30	0.23	0.64	1.38			
Oncogene			BSO	BV6	Combination				
2015,34:4032	Reh	Death	0.03	0.29	0.51	1.62	0.82	Apoptosis	Apoptosis
		Apo	0.03	0.18	0.26	1.33			
Cancer Cell Int			2-DG	Quercetin	Combination				
2016,16:70	HL 60	Death	0.28	0.36	0.90	1.67	2.35	Apoptosis	Apoptosis
		Apo	0.07	0.08	0.57	3.92			
Int J Cancer			Vincristine	BI6727	Combination				
2016,138:497	A4573	Death	0.70	0.46	0.92	1.10	2.01	Apoptosis	Apoptosis
		Apo	0.16	0.18	0.69	2.20			
	SK-ES-1	Death	0.66	0.27	0.86	1.14	1.27	Apoptosis	Apoptosis
		Apo	0.29	0.24	0.66	1.45			
J Exp Clin Cancer Res			LCL161	Paclitaxel	Combination				
2016,35:158	A549	Death	0.08	0.43	0.62	1.30	0.95	Apoptosis	Apoptosis
		Apo	0.14	0.24	0.43	1.24			
	H460	Death	0.09	0.42	0.64	1.34	0.82	Apoptosis	Apoptosis
		Apo	0.12	0.21	0.33	1.10			
J Pharmacol Sci			Allicin	5-fluorouracil	Combination				
2016,131:233	SK-Hep-1	Death	0.03	0.44	0.57	1.25	0.90	Apoptosis	Apoptosis
		Apo	0.02	0.08	0.10	1.13			
	BEL-7402	Death	0.10	0.27	0.46	1.35	0.93	Apoptosis	Apoptosis
		Apo	0.03	0.08	0.14	1.25			
Lasers Med Sci			Melatonin	Light	Combination				
2016,31:1565	HUVEC	Death	0.48	0.04	0.61	1.20	1.31	Apoptosis	Apoptosis
		Apo	0.17	0.09	0.38	1.57			
Oncotarget			Honokiol	Magnolol	Combination				
2016,7:29116	LN229	Death	0.30	0.00	0.53	1.71	2.46	Apoptosis	Apoptosis
		Apo	0.05	0.01	0.25	4.21			
	U87MG	Death	0.39	0.00	0.70	1.78	0.76	Nonapoptosis involved	Apoptosis
		Apo	0.07	0.02	0.12	1.35			
Oncotarget			Chloroquine	Rapamycin	Combination				
2016,7:58075	HA22T	Death	0.08	0.03	0.31	2.73	0.87	Apoptosis	Apoptosis
		Apo	0.12	0.04	0.37	2.38			
			Chloroquine	Vinorelbine	Combination				
	HA22T	Death	0.08	0.23	0.32	1.09	1.23	Apoptosis	Apoptosis
		Apo	0.12	0.16	0.35	1.34			
			Rapamycin	Vinorelbine	Combination				
	HA22T	Death	0.03	0.23	0.32	1.26	1.29	Apoptosis	Apoptosis
		Apo	0.04	0.16	0.32	1.62			
Oncotarget			Shikonin	Arsenic trioxide	Combination				
2016,7:70504	HepG2	Death	0.12	0.19	0.72	2.48	0.60	Nonapoptosis involved	Apoptosis
		Apo	0.15	0.18	0.45	1.49			
	Hep3B	Death	0.11	0.17	0.63	2.40	0.61	Nonapoptosis involved	Apoptosis
		Apo	0.13	0.16	0.39	1.46			
Sci Rep 2016,6:28004			SAHA	TRAIL	Combination				
	MDA-MB-231	Death	0.18	0.13	0.42	1.51	0.74	Nonapoptosis involved	Apoptosis
		Apo	0.22	0.15	0.37	1.11			
	MCF-7	Death	0.20	0.09	0.28	1.04	1.00	Apoptosis	Apoptosis
		Apo	0.23	0.12	0.33	1.04			
Sci Rep 2016;6:38267			Shikonin	5-fluorouracil	Combination				
	SGC-7901	Death	0.01	0.29	0.62	2.03	1.19	Apoptosis	Apoptosis

		Apo	0.05	0.22	0.62	2.41			
			Shikonin	Oxaliplatin	Combination				
	SGC-7901	Death	0.02	0.33	0.58	1.69	1.29	Apoptosis	Apoptosis
		Apo	0.05	0.22	0.57	2.19			
Acta Biochim Biophys			Doxycycline	Doxorubicin	Combination				
Sin 2017,49:999	PC3	Death	0.26	0.38	0.60	1.10	1.18	Apoptosis	Apoptosis
		Apo	0.07	0.12	0.23	1.31			
Apoptosis			Intricatinol	Cisplatin	Combination				
2017,22:1273	A549	Death	0.18	0.26	0.62	1.56	0.82	Apoptosis	Apoptosis
		Apo	0.13	0.28	0.48	1.28			
Biomed Pharmacother			L-arginine	5-fluorouracil	Combination				
2017,88:114	BT-20	Death	0.43	0.46	0.72	1.04	1.12	Apoptosis	Apoptosis
		Apo	0.12	0.12	0.27	1.17			
	MCF-7	Death	0.51	0.79	0.86	0.96	0.78	Nonapoptosis involved	Apoptosis
		Apo	0.23	0.38	0.39	0.75			
Biomed Pharmacother			Apigenin	Cisplatin	Combination				
2017,88:210	$CD44^{+}$ PC3	Death	0.45	0.43	0.59	0.87	1.75	Apoptosis	Apoptosis
		Apo	0.06	0.15	0.31	1.52			
Biomed Pharmacother			TRAIL	Linarin	Combination				
2017,95:1607	U879MG	Death	0.05	0.02	0.52	8.09	5.31	Apoptosis	Apoptosis
		Apo	0.01	0.01	0.69	42.96			
Cell Death Dis			DT-13	Vinorelbine	Combination				
2017,8:e2810	NCI-H460	Death	0.12	0.23	0.55	1.71	1.19	Apoptosis	Apoptosis
		Apo	0.17	0.20	0.69	2.04			
	A549	Death	0.23	0.34	0.60	1.23	2.09	Apoptosis	Apoptosis
		Apo	0.14	0.10	0.59	2.58			
Cell Physiol Biochem			DHA	Curcumin	Combination				
2017,43:589	SKOV3	Death	0.45	0.21	0.61	1.08	0.98	Apoptosis	Apoptosis
		Apo	0.06	0.08	0.14	1.06			
Int J Biochem Cell									
Biol 2017,83:47			TRAIL	Ibulocydine	Combination				
	Hep3B	Death	0.00	0.09	0.46	4.76	1.66	Apoptosis	Apoptosis
		Apo	0.01	0.02	0.20	7.92			
	SK-Hep1	Death	0.07	0.05	0.29	2.57	7.07	Apoptosis	Apoptosis
		Apo	0.01	0.00	0.17	18.21			
Int J Nanomedicine			Actein	Iron oxide	Combination				
2017,12:7627	A549	Death	0.00	0.17	0.34	1.99	0.78	Nonapoptosis involved	Apoptosis
		Apo	0.07	0.11	0.26	1.55			
	H1975	Death	0.00	0.24	0.36	1.44	0.92	Apoptosis	Apoptosis
		Apo	0.06	0.17	0.29	1.32			
Int J Oncol			PEFSO	Doxorubicin	Combination				
2017,50:468	MCF-7	Death	0.38	0.37	0.52	0.85	1.45	Apoptosis	Apoptosis
		Apo	0.37	0.42	0.78	1.23			
Mol Carcinog			Vernodalolr	TRAIL	Combination				
2017,56:2190	Sudhl2	Death	0.18	0.40	0.74	1.47	0.93	Apoptosis	Apoptosis
		Apo	0.07	0.11	0.23	1.36			
Oncotarget			Birinapant	Norcantharidin	Combination				
2017,8:26886	MDA-MD-468	Death	0.11	0.12	0.62	2.89	0.82	Apoptosis	Apoptosis
		Apo	0.10	0.11	0.48	2.36			
Oncotarget			Salinomycin	Gefitinib	Combination				
2017,8:22414	SW1116	Death	0.43	0.32	0.78	1.28	2.30	Apoptosis	Apoptosis
		Apo	0.05	0.09	0.39	2.95			
	HCT-1116	Death	0.19	0.15	0.82	2.64	1.21	Apoptosis	Apoptosis
		Apo	0.04	0.11	0.45	3.20			
Redox Biol			RITA	3-methyladenine	Combination				
2017,13:219	HN4-cisR	Death	0.11	0.35	0.94	2.25	1.06	Apoptosis	Apoptosis
		Apo	0.09	0.26	0.78	2.38			
Sci Rep 2017,7:682			Leucovorin	Bortezomib	Combination				
	HCT-116	Death	0.01	0.35	0.42	1.18	1.07	Apoptosis	Apoptosis
		Apo	0.06	0.39	0.53	1.27			
	HT-29	Death	0.02	0.14	0.25	1.55	0.97	Apoptosis	Apoptosis

		Apo	0.04	0.25	0.43	1.51			
Anticancer Res			Carboplatin	Piroxicam	Combination				
2017,37:1737	T24	Death	0.24	0.01	0.58	2.34	0.27	Nonapoptosis involved	Autophagy
		Apo	0.11	0.10	0.13	0.63			
	5637	Death	0.06	0.01	0.80	10.88	0.07	Nonapoptosis involved	Autophagy
		Apo	0.06	0.08	0.11	0.78			
Oncotarget			Chloroquine	Rapamycin	Combination				
2016,7:58075	Huh7.5.1	Death	0.17	0.33	0.41	0.91	0.41	Nonapoptosis involved	Necroptosis
		Apo	0.06	0.05	0.04	0.37			
			Chloroquine	Vinorelbine	Combination				
	Huh7.5.1	Death	0.17	0.21	0.34	0.98	0.42	Nonapoptosis involved	Necroptosis
		Apo	0.06	0.06	0.05	0.41			
			Rapamycin	Vinorelbine	Combination				
	Huh7.5.1	Death	0.33	0.21	0.47	1.00	0.32	Nonapoptosis involved	Necroptosis
		Apo	0.05	0.06	0.04	0.32			
FEBS Open Bio			Melatonin	Valproic acid	Combination				
2017,7:798	UC3	Death	0.19	0.16	0.28	0.86	0.48	Nonapoptosis involved	Necroptosis
		Apo	0.18	0.12	0.11	0.41			
Cell Death Dis			Siramesine	Lapatinib	Combination				
2016,7:e2307	MDA-MB-231	Death	0.08	0.11	0.61	3.45	0.24	Nonapoptosis involved	Ferroptosis
		Apo	0.11	0.06	0.14	0.82			
Cancer Lett			5-azacytidine	BV6	Combination				
2016,375:127	Tanoue	Death	0.06	0.09	0.34	2.38	0.59	Nonapoptosis involved	Apoptosis + necroptosis
		Apo	0.03	0.10	0.17	1.41			
Cancer Cell Int			Fisetin	Paclitaxel	Combination				
2016,16:10	A549	Death	0.04	0.16	0.48	2.44	0.47	Nonapoptosis involved	Apoptosis + autophagy
		Apo	0.02	0.07	0.10	1.15			
J Pineal Res			Sorafenib	Melatonin	Combination				
2016,61:396	Hep3B	Death	0.06	0.07	0.37	3.11	0.39	Nonapoptosis involved	Apoptosis + autophagy
		Apo	0.27	0.26	0.56	1.20			
J Transl Med			Erlotinib	Sorafenib	Combination				
2016,14:46	GSC11	Death	0.15	0.13	0.69	2.58	0.48	Nonapoptosis involved	Apoptosis + autophagy
		Apo	0.25	0.15	0.45	1.24			
Oncotarget			Bortezomib	Romidepsin	Combination				
2016,7:4454	AGS-BDneo	Death	0.22	0.07	0.88	3.28	0.44	Nonapoptosis involved	Apoptosis + autophagy
		Apo	0.12	0.28	0.53	1.43			
Sci Rep 2016,6:26064			Curcumin	Berberine	Combination				
	MCF-7	Death	0.09	0.28	0.52	1.52	1.18	Apoptosis	Apoptosis + autophagy
		Apo	0.05	0.18	0.40	1.80			
	MDA-MB-231	Death	0.26	0.20	0.51	1.24	1.34	Apoptosis	Apoptosis + autophagy
		Apo	0.12	0.16	0.44	1.66			
Sci Rep 2016,6:34245			DY	5-fluorouracil	Combination				
	HCT-116	Death	0.19	0.30	0.49	1.15	0.69	Nonapoptosis involved	Apoptosis + autophagy
		Apo	0.38	0.33	0.46	0.79			
Oncotarget			Calyxin Y	Cisplatin	Combination				
2017,8:70595	HepG2	Death	0.39	0.33	0.83	1.40	1.32	Apoptosis	Apoptosis + autophagy
		Apo	0.19	0.19	0.63	1.86			
	HepG2/CDDP	Death	0.41	0.08	0.73	1.59	1.86	Apoptosis	Apoptosis + autophagy
		Apo	0.17	0.02	0.55	2.94			
Cancer Lett			BV6	Bortezomib	Combination				
2017,405:63	U-2932	Death	0.09	0.08	0.47	2.92	0.32	Nonapoptosis involved	Apoptosis + necroptosis
		Apo	0.07	0.06	0.12	0.95			
	DG-75	Death	0.15	0.02	0.42	2.59	0.50	Nonapoptosis involved	Apoptosis + necroptosis
		Apo	0.01	0.01	0.02	1.28			

Papers were searched in PubMed using terms of "drug combination, cancer cell, cell death, apoptosis rate, nonapoptotic cell death/ necrosis/ ferroptosis/ necroptosis/ autophagic cell death". Data can be evaluated in 39/198 papers. All percentages (i.e., effects) were >0.

Apo: apoptosis; 2-DG: 2-deoxy-d-glucose; BI 6727: PLK1 inhibitor; BSO: buthionine sulfoximine; BV6/LCL161: Smac mimetic; DHA: dihydroartemisinin; DT-13: saponin monomer 13 of the dwarf lilyturf tuber; DY: extract of Sanguisorba officinalis L. radix; MS275: histone deacetylase inhibitor; PREFO: phenolic extract from flaxseed oil; RITA: reactivation of p53 and induction of tumor cell apoptosis; SAHA: suberoylanilide hydroxamic acid; TRAIL: tumor necrosis factor-related apoptosis-inducing ligand.

Table S2. List of combination indexes (CI) and cell-death modes in released trials (2022−2024)

Trial	Cell	Treatment and effect				CI	CI ratio	Mode based on CI ratio	Mode stated in the trial
Anticancer Agents			Chrysin	Metformin	Combination				
Med Chem	T47D	Death	0.23	0.36	0.65	1.28	1.12	Apoptosis	Apoptosis
2022,22:280		Apo	0.26	0.32	0.71	1.43			
Cells 2022,11:1889			AZD2281	AZD6738	Combination				
	OV-90	Death	0.08	0.39	0.45	1.03	1.00	Apoptosis	Apoptosis
		Apo	0.07	0.15	0.22	1.03			
			AZD2281	MK8776	Combination				
		Death	0.08	0.27	0.34	1.04	0.92	Apoptosis	Apoptosis
		Apo	0.07	0.20	0.25	0.95			
			AZD2281	AZD6738	Combination				
	SKOV-3	Death	0.03	0.37	0.37	0.96	2.39	Apoptosis	Apoptosis
		Apo	0.01	0.04	0.12	2.30			
			AZD2281	MK8776	Combination				
		Death	0.03	0.27	0.31	1.08	1.68	Apoptosis	Apoptosis
		Apo	0.01	0.06	0.13	1.81			
Clin Transl Oncol			Niraparib	Radiation	Combination				
2022,24:1643	KYSE-30	Death	0.08	0.05	0.17	1.35	0.69	Nonapotosis involved	Apoptosis
		Apo	0.10	0.15	0.22	0.93			
	KYSE-150	Death	0.08	0.03	0.15	1.39	0.71	Nonapotosis involved	Apoptosis
		Apo	0.13	0.16	0.27	0.99			
Curr Pharm Des			CG	Cisplatin	Combination				
2022,28:2161	SKOV3	Death	0.24	0.29	0.43	0.93	1.03	Apoptosis	Apoptosis
		Apo	0.19	0.29	0.41	0.96			
Int J Mol Sci			PILA9	Panobinostat	Combination				
2022,23:13019	OVCAR-8	Death	0.28	0.19	0.59	1.42	0.99	Apoptosis	Apoptosis
		Apo	0.28	0.19	0.59	1.40			
	A2780	Death	0.65	0.15	0.78	1.11	1.04	Apoptosis	Apoptosis
		Apo	0.55	0.08	0.68	1.15			
	SKOV3	Death	0.13	0.10	0.44	2.03	0.99	Apoptosis	Apoptosis
		Apo	0.12	0.09	0.39	2.01			
	IGROV-1	Death	0.27	0.26	0.61	1.33	1.09	Apoptosis	Apoptosis
		Apo	0.24	0.21	0.58	1.45			
			MDS 38	Panobinostat	Combination				
	OVCAR-8	Death	0.44	0.47	0.70	1.00	1.01	Apoptosis	Apoptosis
		Apo	0.43	0.45	0.69	1.00			
	A2780	Death	0.41	0.13	0.67	1.38	0.93	Apoptosis	Apoptosis
		Apo	0.38	0.11	0.57	1.28			
	SKOV3	Death	0.13	0.16	0.32	1.19	1.03	Apoptosis	Apoptosis
		Apo	0.12	0.15	0.31	1.22			
	IGROV-1	Death	0.31	0.28	0.53	1.05	1.06	Apoptosis	Apoptosis
		Apo	0.28	0.24	0.50	1.11			
			MDS 42	Panobinostat	Combination				
	OVCAR-8	Death	0.54	0.47	0.75	0.99	1.01	Apoptosis	Apoptosis
		Apo	0.53	0.45	0.74	1.00			
	A2780	Death	0.44	0.13	0.69	1.35	1.15	Apoptosis	Apoptosis
		Apo	0.35	0.11	0.65	1.55			
	SKOV3	Death	0.25	0.24	0.45	1.05	1.00	Apoptosis	Apoptosis
		Apo	0.23	0.23	0.42	1.04			
	IGROV-1	Death	0.35	0.13	0.51	1.17	1.02	Apoptosis	Apoptosis
		Apo	0.30	0.11	0.45	1.20			
			MDS 45	Panobinostat	Combination				
	OVCAR-8	Death	0.04	0.28	0.46	1.49	0.98	Apoptosis	Apoptosis
		Apo	0.04	0.28	0.45	1.46			
	A2780	Death	0.04	0.11	0.22	1.51	1.15	Apoptosis	Apoptosis
		Apo	0.02	0.07	0.16	1.74			
	SKOV3	Death	0.23	0.10	0.57	1.86	1.04	Apoptosis	Apoptosis
		Apo	0.21	0.10	0.54	1.93			
	IGROV-1	Death	0.35	0.13	0.50	1.15	0.99	Apoptosis	Apoptosis
		Apo	0.31	0.11	0.44	1.14			
Int J Mol Sci			Escitalopram	5-fluorouracil	Combination				
2022,23:16179	SNU-1	Death	0.76	0.65	0.88	0.96	1.35	Apoptosis	Apoptosis

		Apo	0.40	0.12	0.61	1.29			
J Cancer Res Clin			Ad-VT	Etoposide	Combination				
Oncol 2022,148:1073	NCI-H446	Death	0.43	0.26	0.58	1.00	1.08	Apoptosis	Apoptosis
		Apo	0.32	0.10	0.42	1.08			
J Pharm Pharmacol			Gemcitabine	HY-PDT	Combination				
2022,74:596	Capan-2	Death	0.33	0.21	0.78	1.66	0.90	Apoptosis	Apoptosis
		Apo	0.05	0.04	0.13	1.49			
Photodiagnosis			3-methyladenine	Oxaliplatin	Combination				
Photodyn Ther	SW620	Death	0.04	0.42	0.52	1.17	0.69	Nonapoptosis involved	Apoptosis
2022,40:103082		Apo	0.11	0.14	0.19	0.81			
	SW620/L-OHP	Death	0.01	0.57	0.64	1.11	0.83	Apoptosis	Apoptosis
		Apo	0.13	0.15	0.24	0.92			
Anticancer Agents			Telaglenastat	EGCG	Combination				
Med Chem	KM3/BTZ	Death	0.28	0.19	0.70	1.68	1.05	Apoptosis	Apoptosis
2023,23:779		Apo	0.04	0.10	0.24	1.76			
Appl Biochem			Berberiner-PDT	Valproic acid	Combination				
Biotechnol	MDA-MB-231	Death	0.42	0.01	0.46	1.08	2.41	Apoptosis	Apoptosis
2023,195:6752		Apo	0.13	0.06	0.48	2.60			
BMC Complement			Protocatechuic aldehyde	Dacarbazine	Combination				
Med Ther 2023,23:111	A375	Death	0.66	0.82	0.95	1.01	1.44	Apoptosis	Apoptosis
		Apo	0.17	0.10	0.37	1.45			
	SK-MEL-28	Death	0.28	0.52	0.73	1.12	0.97	Apoptosis	Apoptosis
		Apo	0.04	0.16	0.21	1.08			
Cell Cycle			Trifluorothymidine	Cryptotanshinone	Combination				
2023,22:1463	HGC-27	Death	0.28	0.48	0.68	1.09	1.12	Apoptosis	Apoptosis
		Apo	0.19	0.09	0.32	1.22			
		Death	0.50	0.48	0.76	1.03	1.23	Apoptosis	Apoptosis
		Apo	0.29	0.09	0.45	1.27			
Clin Transl Oncol			Radiation	Chrysin	Combination				
2023,25:2559	MDA-MB-231	Death	0.28	0.45	0.75	1.24	0.90	Apoptosis	Apoptosis
		Apo	0.35	0.45	0.72	1.12			
Int J Mol Sci			Z-ligustilide	Cisplatin	Combination				
2023,24:17046	A549/DDP	Death	0.06	0.05	0.34	3.18	0.89	Apoptosis	Apoptosis
		Apo	0.02	0.02	0.10	2.84			
	H460/DDP	Death	0.03	0.12	0.35	2.39	1.95	Apoptosis	Apoptosis
		Apo	0.03	0.02	0.22	4.66			
J Enzyme Inhib Med			HCPT	Crizotinib	Combination				
Chem 2023,38:1	H460	Death	0.41	0.27	0.61	1.06	1.45	Apoptosis	Apoptosis
		Apo	0.22	0.05	0.39	1.54			
	HCC827	Death	0.14	0.31	0.53	1.30	0.84	Apoptosis	Apoptosis
		Apo	0.27	0.13	0.40	1.09			
J Pharm Pharmacol			Jigengtang	Cisplatin	Combination				
2023,75:784	A549/DDP	Death	0.55	0.01	0.89	1.61	0.69	Nonapoptosis involved	Apoptosis
		Apo	0.56	0.12	0.68	1.11			
Mol Biol Rep			Calcitriol	Cisplatin	Combination				
2023,50:697	T24	Death	0.21	0.18	0.44	1.25	0.59	Nonapoptosis involved	Apoptosis
		Apo	0.16	0.09	0.17	0.73			
Naunyn			SIX4-siRNA	Temozolomide	Combination				
Schmiedebergs Arch	A-172	Death	0.06	0.26	0.40	1.31	1.39	Apoptosis	Apoptosis
Pharmacol		Apo	0.09	0.24	0.56	1.82			
2023,396:2741									
		Death	0.06	0.50	0.67	1.26	1.05	Apoptosis	Apoptosis
		Apo	0.09	0.48	0.70	1.33			
BioMed Res Int			5-fluorouracil	Thymoquinone					
2024,2024,6231095	SW-480	Death	0.48	0.48	0.71	0.97	1.15	Apoptosis	Apoptosis
		Apo	0.65	0.18	0.8	1.12			
Bioorg Chem			Liriopesides B	Gemcitabine	Combination				
2024,142:106937	Panc-1	Death	0.17	0.12	0.55	2.04	0.76	Nonapoptosis involved	Apoptosis
		Apo	0.13	0.17	0.43	1.55			
Biotechnol Appl			Hederagenin	28-Glc-hederagenin	Combination				
Biochem 2024,71:979	HeLa	Death	0.73	0.35	0.99	1.20	0.52	Nonapoptosis involved	Apoptosis
		Apo	0.23	0.16	0.22	0.63			
BMC Cancer			Brequinar	Venetoclax	Combination				
2024,24:761	SU-DHL4	Death	0.34	0.08	0.49	1.25	0.68	Nonapoptosis involved	Apoptosis
		Apo	0.14	0.11	0.20	0.84			

Chem Biol Drug Des			TRAIL	Cisplatin	Combination				
2024,103:e14357	OVCAR3	Death	0.09	0.17	0.29	1.19	0.79	Nonapoptosis involved	Apoptosis
		Apo	0.33	0.47	0.60	0.93			
	Caov3	Death	0.17	0.32	0.46	1.06	1.20	Apoptosis	Apoptosis
		Apo	0.27	0.31	0.63	1.27			
	CR-Caov3	Death	0.20	0.03	0.24	1.07	1.11	Apoptosis	Apoptosis
		Apo	0.25	0.14	0.42	1.19			
Drug Dev Res			EGCG	Apatinib	Combination				
2024,85:e22239	H1299	Death	0.42	0.26	0.56	0.98	1.22	Apoptosis	Apoptosis
		Apo	0.13	0.11	0.27	1.20			
Drug Dev Res			Ixazomib	A7	Combination				
2024,85:e70016	MM1.S	Death	0.42	0.25	0.55	0.97	1.34	Apoptosis	Apoptosis
		Apo	0.60	0.07	0.81	1.30			
			Ixazomib	A13	Combination				
		Death	0.46	0.02	0.44	0.94	0.91	Apoptosis	Apoptosis
		Apo	0.60	0.03	0.52	0.86			
			Ixazomib	B15	Combination				
		Death	0.38	0.28	0.58	1.04	0.87	Apoptosis	Apoptosis
		Apo	0.60	0.06	0.55	0.90			
			Ixazomib	B26	Combination				
		Death	0.44	0.38	0.56	0.86	1.32	Apoptosis	Apoptosis
		Apo	0.60	0.06	0.70	1.14			
Environ Toxicol			1G6-D7	Olaparib	Combination				
2024,39:4884	A549	Death	0.17	0.57	0.74	1.15	0.67	Nonapoptosis involved	Apoptosis
		Apo	0.11	0.11	0.16	0.77			
J Biochem Mol			Tilianin	Sufentanil	Combination				
Toxicol	A549	Death	0.20	0.24	0.53	1.35	0.82	Apoptosis	Apoptosis
2024,38:e23761		Apo	0.19	0.22	0.41	1.11			
	H1299	Death	0.18	0.22	0.49	1.36	0.64	Nonapoptosis involved	Apoptosis
		Apo	0.22	0.25	0.36	0.87			
J Oleo Sci			Rg3	Rg5	Combination				
2024,73:219	A549	Death	0.37	0.48	0.76	1.13	1.21	Apoptosis	Apoptosis
		Apo	0.28	0.35	0.73	1.37			
Probiotics Antimicrob			Rhamnosin	Lysostaphin	Combination				
Proteins 2024,16:713	KKU-213A	Death	0.29	0.17	0.56	1.36	0.74	Nonapoptosis involved	Apoptosis
		Apo	0.14	0.30	0.40	1.01			
	KKU-213A-GR	Death	0.41	0.16	0.83	1.65	0.41	Nonapoptosis involved	Apoptosis
		Apo	0.30	0.36	0.37	0.67			
	KKU-213B	Death	0.29	0.28	0.84	1.72	2.34	Apoptosis	Apoptosis
		Apo	0.01	0.02	0.12	4.03			
	KKU-213B-GR	Death	0.20	0.21	0.80	2.17	0.48	Nonapoptosis involved	Apoptosis
		Apo	0.05	0.09	0.14	1.03			
Toxicol In Vitro			Cu-Cy NP	Radiation	Combination				
2024,99:105878	Hela	Death	0.07	0.09	0.19	1.24	1.31	Apoptosis	Apoptosis
		Apo	0.06	0.10	0.25	1.62			
			Cisplatin	Radiation	Combination				
		Death	0.17	0.09	0.39	1.59	0.61	Nonapoptosis involved	Apoptosis
		Apo	0.15	0.10	0.23	0.98			
			Cu-Cy NP + cisplatin	Radiation	Combination				
		Death	0.46	0.09	0.58	1.14	1.08	Apoptosis	Apoptosis
		Apo	0.36	0.10	0.52	1.23			
Mol Biol Rep			Berberine	APP	Combination				
2025,52,37	PC-9	Death	0.28	0.10	0.33	0.94	1.33	Apoptosis	Apoptosis
		Apo	0.69	0.09	0.90	1.25			
Med Oncol 2024,41:31			LY294002	Tubastatin A	Combination				
	CMK	Death	0.39	0.42	0.58	0.90	0.42	Nonapoptosis involved	Autophagy
		Apo	0.07	0.08	0.05	0.37			
			LY294002	Tubastatin A	Combination				
	MOLM-13	Death	0.35	0.20	0.45	0.94	0.77	Nonapoptosis involved	Autophagy
		Apo	0.05	0.09	0.10	0.72			
			LY294002	Vorinostat	Combination				
		Death	0.34	0.22	0.53	1.09	0.72	Nonapoptosis involved	Autophagy
		Apo	0.05	0.11	0.12	0.79			

Int J Pharm			MJNP	Hyperthermia	Combination				
2024,653:123888	C6	Death	0.11	0.28	0.63	1.75	0.93	Apoptosis	Apoptosis + necrosis
		Apo	0.07	0.03	0.16	1.63			
	OLN-93	Death	0.07	0.19	0.55	2.23	1.07	Apoptosis	Apoptosis + necrosis
		Apo	0.15	0.03	0.42	2.39			
			Quercetin/5-fluorouracil/MJNP	Hyperthermia	Combination				
	C6	Death	0.38	0.28	0.87	1.57	1.03	Apoptosis	Apoptosis + necrosis
		Apo	0.07	0.17	0.37	1.62			
	OLN-93	Death	0.36	0.19	0.74	1.54	0.78	Nonapoptosis involved	Apoptosis + necrosis
		Apo	0.25	0.17	0.45	1.19			
Pathol Res Pract			SIX4 siRNA	Cisplatin	Combination				
2022,240:154194	A-375	Death	0.11	0.50	0.95	1.71	0.97	Apoptosis	Apoptosis + autophagy?
		Apo	0.27	0.14	0.61	1.66			
Free Radic Biol Med			Falnidamol	Cisplatin	Combination				
2022,183:106	A549	Death	0.54	0.50	0.75	0.97	1.68	Apoptosis	Apoptosis + ferroptosis
		Apo	0.11	0.11	0.34	1.64			
	PC-9	Death	0.52	0.57	0.78	0.98	1.59	Apoptosis	Apoptosis + ferroptosis
		Apo	0.13	0.16	0.42	1.56			
Anticancer Agents			Apigenin	AA	Combination				
Med Chem	LnCap	Death	0.49	0.40	0.60	0.86	0.83	Apoptosis	Apoptosis + nonapoptosis
2022,22:3148		Apo	0.27	0.26	0.33	0.72			

Papers were searched in PubMed using terms of "drug combination, cancer cell, cell death, apoptosis rate, nonapoptotic cell death/ necrosis/ ferroptosis/ cuproptosis/ necroptosis/ pyroptosis/ autophagic cell death". Data can be evaluated in 37/308 papers.

Apo: apoptosis; 1G6-D7: eHSP90α mcAb; A7/A13/B15/B26: SIRT3 inhibitor; AA: abiraterone acetate; Ad-VT: oncolytic adenovirus; APP: atmospheric pressure plasma; AZD2281: PARP inhibitor; AZD6738: ATR inhibitor; CG: calycosin-7-O-β-D-glucoside; Cu-Cy NP: copper-cysteamine nanoparticles; EGCG: epigallocatechin-3-gallate; HCPT: 10-hydroxycamptothecin; HY-PDT: hypericin-photodynamic therapy; LY294002: PI3K inhibitor; MDS: microtubule destabilizing sulfonamides; MJNP: magnetic nanoparticles; MK8776: CHK1 inhibitor; PILA9: microtubule destabilizing agent; NP: nanoparticle; Rg: ginsenosides; TRAIL: tumor necrosis factor-related apoptosis-inducing ligand.
